# Dynamic Stretching Increases the Eccentric Rate of Force Development, but not Jump Height in Female Volleyball Players

**DOI:** 10.2478/hukin-2022-0071

**Published:** 2022-11-08

**Authors:** Mauricio Araya-Ibacache, Esteban Aedo-Muñoz, Pablo Carreño-Ortiz, Christopher Moya-Jofré, Amaya Prat-Luri, Hugo Cerda-Kohler

**Affiliations:** 1 Applied Sports Science Unit, High-Performance Center, Instituto Nacional de Deportes, Santiago Chile; 2 Kinesiology Departament, Hospital del Trabajador, Trabajador Chile; 3 Postgraduate Program in Physical Education, School of Physical Education and Sports, Federal University of Rio de Janeiro, Rio de Janeiro, Brazil; 4 Physical Activity, Sport and Health Laboratory, Universidad de Santiago, Santiago Chile; 5 Physical Education, Universidad Santo Tomás, Santiago, Chile; 6 Sport Research Centre, Universidad Miguel Hernández, Hernández España; 7 Exercise Sciences Laboratory, Integrative Exercise Physiology Section, Clínica MEDS, Chile; 8 Department of Physical Education, Sports and Recreation, Universidad Metropolitana de Ciencias de la Educación, Santiago Chile; 9 Escuela de Ciencias del Deporte y Actividad Física, Facultad de Salud, Universidad Santo Tomás, Tomás Chile

**Keywords:** countermovement jump, RFD, volleyball, stretching, performance

## Abstract

The present study aimed to analyze the effect of static and dynamic stretching exercises on the rate of force development (RFD) during the eccentric braking phase and jump height in a countermovement jump (CMJ) in female volleyball players. Thirty female volleyball players were randomly distributed in a static stretching (n = 10; SG), a dynamic stretching, and (n = 10; DG) a control group (n = 10; CG). A force plate and a 3D analysis system were employed to detect the eccentric braking phase during the CMJ. The RFD was analyzed in RFD (RFDi) intervals and the accumulated RFD (RFDa), and normalized to body mass. The SG experienced a likely small decrease in the RFDa (mean difference −17.4 N/s/kg) and a likely small decrease in the RFDi (mean difference −19.1 N/s/kg). Contrarily, the DG showed a likely small increase in the RFDa (mean difference 31.2 N/s/kg) and a most likely small increase in the RFDi (mean difference 34.8 N/s/kg). The effect of both static and dynamic stretching on jump height was trivial. Practitioners should consider utilizing dynamic stretching exercises instead of static stretching before a competition in female volleyball players. Further research is needed in order to find complementary strategies during the warm-up that could increase jump height.

## Introduction

A warm-up before a competition is essential for preparing athletes for the main activity. Warm-up protocols differ depending on the sports characteristics and should include sport-specific movement patterns to optimize the benefits. Static stretching has been largely considered an essential part of warm-ups. Nevertheless, its effectiveness before a competition in explosive sports has been questioned since it is related to a negative impact on performance, especially on muscular power and strength (Ciemiński, 2017; Cramer et al., 2005; Power et al., 2004), jump performance (Power et al., 2004), and sprint ability (Winchester et al., 2008). On the other hand, dynamic stretching has been recommended for sports requiring dynamic and explosive actions, showing to improve performance and influencing the range of movement for the sport (Galazoulas, 2017). Vertical jump tests that involve the stretch-shortening cycle seem to reflect the effect on power and strength-related variables precisely (Driss et al., 2015). Accordingly, time-force variables such as the rate of force development (RFD) could be analyzed, reflecting when the maximal peak of force has been reached. An association between the vertical displacement and the peak RFD has been reported (McLellan et al., 2011) and is considered an essential indicator of explosive strength (Taber et al., 2016). However, scarce literature analyzes the eccentric phase, which could be a better predictor of jump performance in a countermovement jump (Laffaye and Wagner, 2013). Also, rapid restabilization of joints following a mechanical perturbation (e.g., landing from a jump) is essential to prevent injury, where the eccentric RFD could be considered functionally more relevant than peak force for this purpose (Buckthorpe and Roi, 2017). Furthermore, it is essential to note differences in analyzing the RFD, comparing the RFD at intervals (*i.e*., the peak of force determined by time intervals) or accumulated (i.e., time to reach the peak of force), which could improve the comparison among studies (Laffaye and Wagner, 2013).

The study aimed to analyze the acute effects of static and dynamic stretching exercises on the RFD during the eccentric braking phase and jump height in a countermovement jump (CMJ) in female volleyball players. It was hypothesized that dynamic stretching exercises would increase the RFD during the eccentric braking phase in a CMJ, with no changes in jump height.

## Methods

### Participants

Thirty female volleyball players (age: 21.06 ± 2.15 years; body height: 167.6 ± 6.9 cm; body mass: 65.96 ± 8.6 kg) participated voluntarily in the study. Before participation, all players gave their written informed consent. The inclusion criteria were as follows: a regular physical activity of at least 30 minutes, three times a week, and no history of any injury of the lower body or lower back disease in the past year and during the experiment. The study was approved by the ethics committee of the Santo Tomás University of Chile, N^⍛^ 40.18.

### Design and Procedures

The assessments took place at the Sports Biomechanics Laboratory of the High-Performance Center of Chile. All participants performed the same warm-up protocol before the jump test: (i) 5 min cycling on a cycloergometer at 30 rpm, (ii) four progressive CMJs on a force plate with 30 s rest intervals between each repetition. Afterwards, participants were divided into three groups: 1) a static stretching group (SG); 2) a dynamic stretching group (DG); and 3) a control group (CG). The two experimental groups (SG and DG) performed stretching exercises after the general warm-up, and the CG did not perform any other exercise before the jump test. The two stretching protocols included the same muscular groups in the subsequent order: 1) hip adductors; 2) hamstrings; 3) tensor fasciae latae; 4) rectus femoris, and 5) triceps surae. The SG performed five repetitions of 30 s for each muscle, with a 10 s rest interval between each repetition. The DG performed three bouts of wide-range repetitive movements. Each bout was 10 s long, with a 10-s rest interval between each cycle. A practitioner and an assistant controlled the correct execution of the different stretches. These muscular groups were chosen because of their involvement in the vertical jump. Participants were advised to perform stretching until feeling a bit of “tightness”, but without pain. All participants performed a single testing session. They performed four countermovement jump (CMJ) tests, with 3 min recovery between subsequent jumps and before and after the stretching exercises in the experimental groups. The jumps were performed on a force plate (Bertec 4060-07, Columbus, OH). Furthermore, a 3D analysis system (Vicon VL 0390) registered the kinematics of the jumps, for which a lower-limb model was employed (Vicon Motion Systems, 2019). Both force plates and the Vicon system were formerly calibrated.

### Measures

Force-related variables were obtained from the two force plates and sampled at 1000 Hz. Displacement, acceleration, and speed were assessed with a 10-camera optoelectronic system. The cameras sampled at 200 Hz (V5, Vicon, England) and registered the vertical displacement of the sacral marker ubicated at L4-L5 level, just above the sacrum (Figure 2B). The start and the end of the CMJ were likewise established through the speed of this marker. The start was considered the negative peak of speed. Concurrently, the end was set when the speed changed from negative to positive (McMahon et al., 2018), which was confirmed by the lowest vertical position of the sacrum marker (Figure 2A). The peak of the RFD was analyzed in the time window of the eccentric braking phase through two different methods: a) the interval RFD (RFDi), in which the peak of the RFD was calculated each 10 ms, allowing a more specific response of the RFD depending on the interval; b) the accumulated RFD (RFDa), where the analysis was applied to specific accumulated time bands of 10 ms (*i.e*., ranging 0–10 to 0–250 ms) (Haff et al., 2015). The RFD selection for statistics analysis was RFDi peak and RFDa peak; these values were normalized to the body mass of participants.

**Figure 1:**
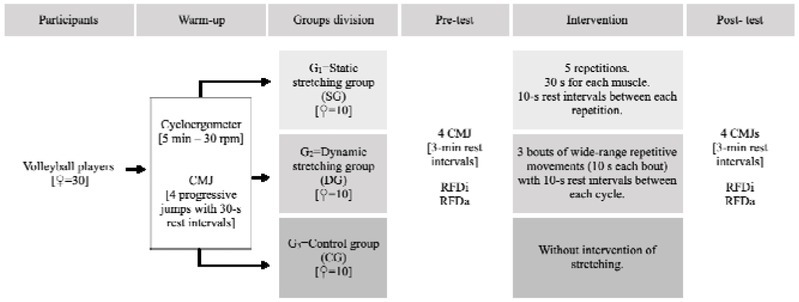
Design and procedures. CMJ: countermovement jump; G: group; RFDi: interval rate of force development; RFDa: accumulated rate of force development.

**Figure 2:**
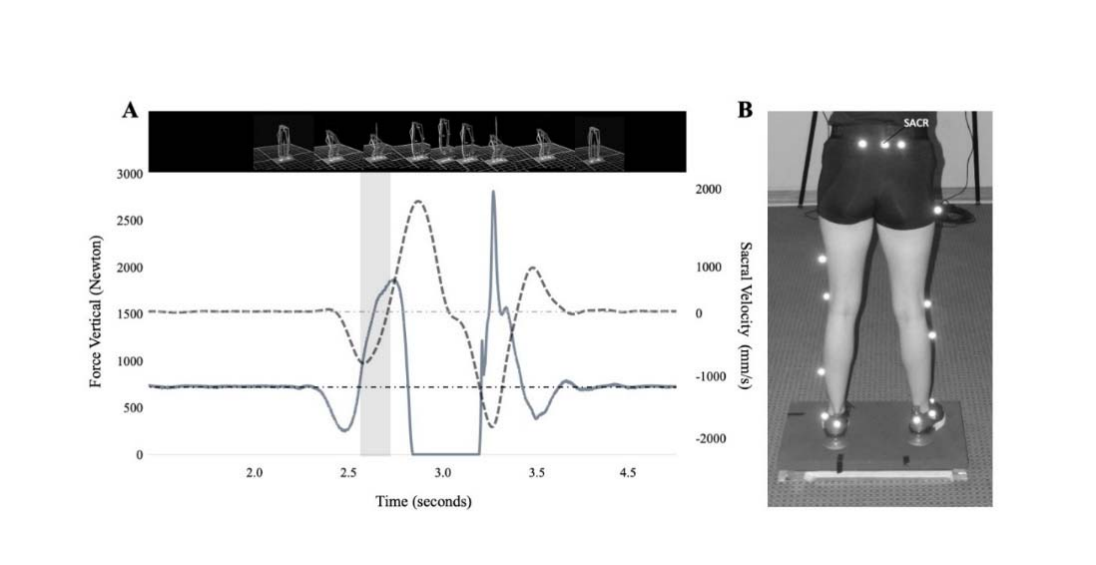
A) Zone of analysis of the CMJ for the interval and the accumulated rate of force development in the eccentric braking phase. Sacral Velocity: discontinues line; Vertical Force: continuous line; Gray Zone: eccentric braking phase. B) Lower body modeling with Plug-in Gate and a sacral marker. SACR: Sacral marker.

### Statistical Analysis

Data and figures are presented as mean ± standard deviation (SD) and 90% confidence limits/confidence intervals (CL/CI). All data were first log-transformed to reduce bias arising from non-uniformity error. Changes between pre-post values in the rate of force development and jump height were assessed using standardized differences based on the Cohen’s effect-size principle. Then, magnitude-based inference (MBI) was used as an equivalent of Bayesian with a minimally informative prior distribution. Probabilities were used to make a qualitative probabilistic mechanistic inference about the true changes/differences in changes within the groups, which were assessed compared to the smallest worthwhile change (0.2 x pre-tests between-subjects SDs) (Buchheit, 2016). Thresholds values for standardized changes/differences in the changes were >0.2 (small), >0.6 (moderate), >1.2 (large) and >2 (very large) (Lacome et al., 2019). Finally, the probability of any substantial difference relative to the predefined target values was interpreted using the following scale: <0.5%, most unlikely; 0.5–5%, very unlikely; 5–25%, unlikely; 25–75%, possibly; 75–95%, likely; 95– 99.5%, very likely; >99.5%, most likely (Batterham and Hopkins, 2006). The effects were declared relevant if the outcome probability was likely (75%). Pearson’s product-moment correlation analysis was used to compare the association between the RFD and jump height. The following criteria were adopted to interpret the magnitude of the correlation (r) between variables: ≥0.1 trivial, >0.1–0.3 small, >0.3–0.5 moderate, >0.5–0.7 large, >0.7–0.9 very large, and >0.9–1.0 almost perfect. If 90% confidence intervals overlapped small positive and negative values, the magnitude was deemed unclear; otherwise, the magnitude was deemed the observed magnitude. Statistical significance was set at *p* < 0.05. The analysis was performed using the “mbir” package of the R software.

## Results

### Accumulative analysis of the rate of force development

**Figure 3:**
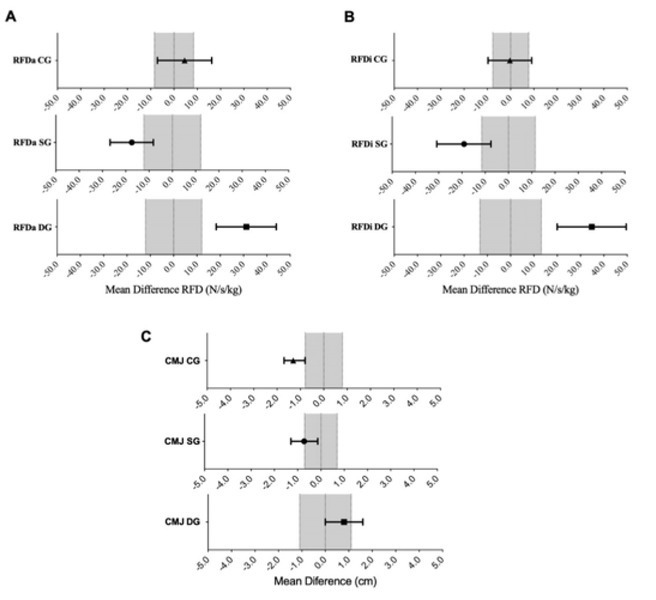
Differences on the A) accumulative analysis, B) interval analysis of the eccentric rate of force development, and C) jump height between the SG, DG, and CG before and after the flexibility intervention. RFD: rate of force development; RFDa: accumulated rate of force development; RFDi: interval rate of force development; CG: control group; SG: static stretching group; DG: dynamic stretching group.

**Figure 4:**
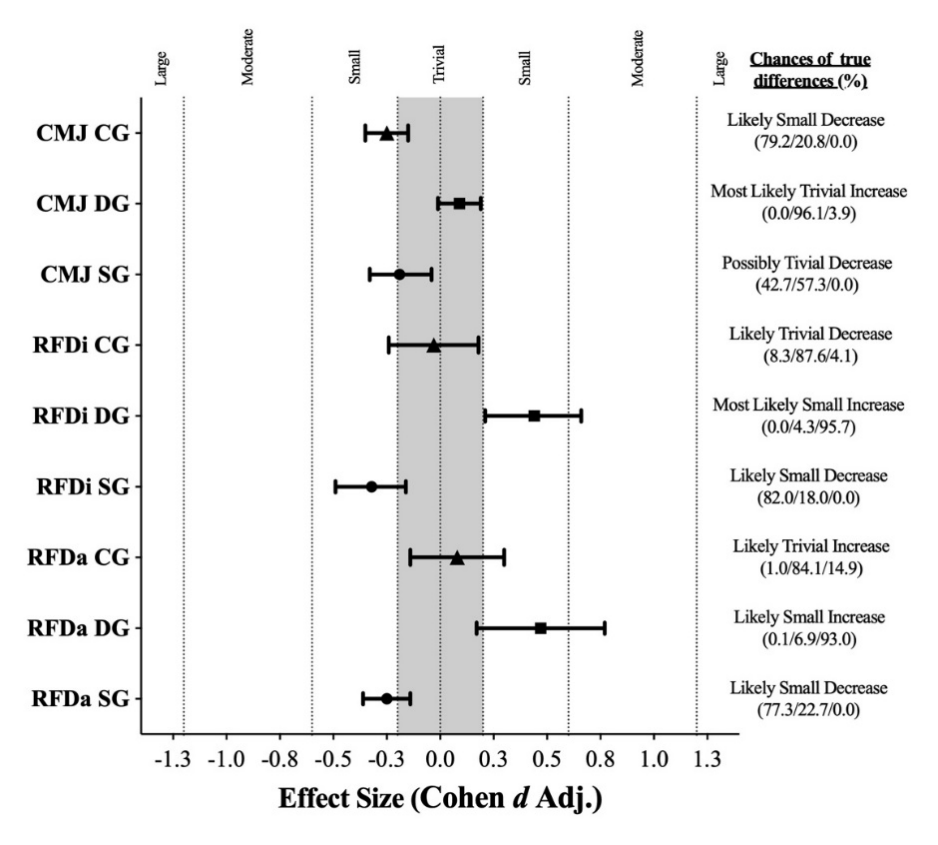
Differences as effect size in the analysis of the rate of force development and jump height between the SG, DG, and CG before and after the flexibility intervention. CMJ: countermovement jump; RFDa: accumulated rate of force development; RFDi: interval rate of force development; CG: control group; SG: static stretching group; DG: dynamic stretching group.

Analysis showed a likely small decrease in the accumulative RFD (mean difference −17.4 N/s/kg, 90% CL (-26.7; -8.1); *d* = -0.25; *p* = 0.003; Figure 3A and Figure 4) for the static stretching group, while for the dynamic stretching group, results showed a likely small increase (mean difference 31.2 N/s/kg, 90% CL (18.3; 44.1); *d* = 0.47; *p* = 0.019; Figure 3A and Figure 4). Analysis for the control group showed a likely trivial increase and no significant difference (mean difference 4.5 N/s/kg, 90% CL (-7.1; 16.2); *d* = 0.08; *p* = 0.531; Figure 3A and Figure 4).

### Interval analysis of the rate of force development

The results showed a likely small decrease in the interval RFD (mean difference −19.1 N/s/kg, 90% CL (-30.8; -7.5); *d* = -0.32; *p* = 0.006; Figure 3B and Figure 4) for the static stretching group. For the dynamic stretching group, results showed a most likely small increase in the interval RFD (mean difference 34.8 N/s/kg, 90% CL (20.0; 49.6); *d* = 0.44; *p* = 0.006; Figure 3B and Figure 4), while the results for the control group showed a likely trivial decrease and no significant difference (mean difference -0.31 N/s/kg, 90% CL (-9.7; 9.0); *d* = -0.03; *p* = 0.825; Figure 3B and Figure 4).

### Jump height analysis

The results showed a possibly trivial decrease in the CMJ (mean difference −0.72 cm, 90% CL (-1.29; -0.14); *d* = -0.19; *p* = 0.039; Figure 2C and Figure 3) for the static stretching group. For the dynamic stretching group, however, results show a most likely trivial increase and no significant difference (mean difference 0.80 cm, 90% CL (0.00; 1.60); *d* = 0.09; *p* = 0.119; Figure 3C and Figure 4). The results for the control group showed a likely small decrease in the CMJ (mean difference -1.31 cm, 90% CL (-1.70; -0.80); *d* = -0.25; *p* = 0.002; Figure 3C and Figure 4).

### Correlation between the RFD and jump height

When considering all data from the three groups, there was an unclear correlation between jump height and both RFDa (r = 0.17, 90% CL (0.15; 0.45); *p* = 0.382) and RFDi (r = 0.21, 90% CL (0.10; 0.49); *p* = 0.256).

## Discussion

The following study aimed to compare the impact of two stretching methods (static and dynamic) on the RFD of the eccentric braking phase and jump height in a CMJ in female volleyball players. The results confirm our hypothesis, showing that static stretching produced a decrease in explosive strength, whereas dynamic stretching increased this variable. Additionally, no stretching (control group) showed no changes in the RFD. No changes were found for jump height.

Explosive-force variables highly influence performance in volleyball; however, little evidence exists considering the acute effects of different types of stretching in female athletes. Several studies have compared the effects of static and dynamic stretching exercises on lower-limb power in men, finding increases in dynamic stretching groups, yet decreases in static stretching groups (Galazoulas, 2017; Hough et al., 2009). All these outcomes are consistent with our results. Increased power observed after dynamic stretching could be influenced because of the higher muscle temperature reached after stretching or increased action potentials, leading to a higher number of crossed bridges (Houston and Grange, 1990). Contrarily, static stretching could be influenced by reduced stiffness, changes in the muscular length and tension applied, and different stretching reflex thresholds (Nelson et al., 2001).

Regarding eccentric-force variables, Cramer et al. (2005) assessed the acute effects of static stretching on the eccentric torque during an isokinetic protocol. However, they did not find any changes in force values. The nature of the test could influence the results because stretch-shortening demands in a jump differ from an isokinetic task. Our results support this evidence since the dynamic stretching group showed an increment likewise in the peak RFD. In contrast, the peak RFD in the static stretching group was reduced. These results are in agreement with those previously found by Oliveira et al. (2016) who observed a decrease in the RFD after static stretching. Also, Kruse et al. (2015) found substantial improvements in the average RFD and peak force after dynamic stretching compared to static stretching. These findings seem to support the idea that the RFD is a suitable predictor of performance in sports characterized by high levels of acceleration (Marques et al., 2008).

It is postulated that greater flexibility is not associated with a reduction in total injuries (Thacker et al., 2004). However, the increased eccentric RFD stimulated by dynamic stretching could prevent injuries as rapid re-stabilization of joints following a mechanical perturbation (*e.g*., landing from a jump) which is essential to prevent injury (Buckthorpe and Roi, 2017). Nevertheless, further research is needed in order to confirm this hypothesis. Finally, we found no substantial effects of stretching on jump height, which partially agrees with others studies (Bradley et al., 2007; Christensen and Nordstrom, 2008). Our results show no relationship between the eccentric RFD and jump height, highlighting the importance of establishing complementary strategies that potentiate jump performance. In practical terms, our results indicate that:

– the RFD during the eccentric braking phase in a countermovement jump (CMJ) is adversely affected by static stretching by ~11% and improved after dynamic stretching by ~23-29%.
– the interval RFD is more sensitive than accumulative RFD in detecting acute changes caused by stretching.
– the acute effects of both static and dynamic stretching on jump height are trivial. Practitioners should find complementary strategies during a warm-up in order to increase jump height.
– dynamic stretching could reduce injury risk, providing a protective mechanism for joint re-stabilization.

The main limitation of the study was that participants came from different teams and were probably submitted to different training systems. Nevertheless, this study aimed to evaluate the acute effects of stretching from a baseline condition of each athlete. However, this may have influenced the variety of the RFD observed in the results. Another limitation that should be considered in future research is more precise stretching protocol that could be evaluated and reproduced.

## Conclusions

In many sports disciplines, the ability to generate force quickly is strongly related to performance and injury risk. The RFD has proved to be an excellent indicator of explosive force. Our study evaluated the acute effects of different stretching routines (static and dynamic) on the RFD during the eccentric braking phase of a countermovement jump. This study showed that static-type stretching exercises produced a decrease in the post-stretching RFD. In contrast, the dynamic stretching routine increased the RFD. Also, the force-time curve opens new study perspectives to search for the most reliable methods/tests to determine the different variables involved and how these variables could be related to injury risk/prevention. From a practical point of view, we suggest incorporating dynamic stretching exercises instead of static stretching before a competition in female volleyball players.
